# Mitochondrial a Kinase Anchor Proteins in Cardiovascular Health and Disease: A Review Article on Behalf of the Working Group on Cellular and Molecular Biology of the Heart of the Italian Society of Cardiology

**DOI:** 10.3390/ijms23147691

**Published:** 2022-07-12

**Authors:** Roberta Paolillo, Stefania D’Apice, Gabriele Giacomo Schiattarella, Pietro Ameri, Domenica Borzacchiello, Daniele Catalucci, Cristina Chimenti, Lia Crotti, Sebastiano Sciarretta, Daniele Torella, Antonio Feliciello, Cinzia Perrino

**Affiliations:** 1 Department of Advanced Biomedical Sciences, Federico II University, 80131 Naples, Italy; robe.paolillo@gmail.com (R.P.); stedapice@gmail.com (S.D.); gabrielegiacomo.schiattarella@unina.it (G.G.S.); 2 Department of Internal Medicine, University of Genova, 16132 Genova, Italy; pietro.ameri@unige.it; 3 Department of Molecular Medicine and Medical Biotechnology, Federico II University, 80131 Naples, Italy; domenica.borzacchiello@unina.it (D.B.); feliciel@unina.it (A.F.); 4 Institute of Genetic and Biomedical Research (IRGB), National Research Council (CNR), Milan Unit, 20133 Milan, Italy; daniele.catalucci@cnr.it; 5 Istituto di Ricovero e Cura a Carattere Scientifico, Humanitas Research Hospital, 20089 Milan, Italy; 6 Department of Clinical Medicine, Anesthesiology and Cardiovascular Sciences, University of Rome “La Sapienza”, 00161 Rome, Italy; cristina.chimenti@uniroma1.it; 7 Department of Medicine and Surgery, University of Milano-Bicocca, 20126 Milan, Italy; liacrotti@yahoo.it; 8 Department of Cardiology, Istituto di Ricovero e Cura a Carattere Scientifico, Istituto Auxologico Italiano, 20149 Milan, Italy; 9 Department of Medical and Surgical Sciences and Biotechnologies, University of Rome “La Sapienza”, 04100 Rome, Italy; sebastiano.sciarretta@uniroma1.it; 10 Istituto di Ricovero e Cura a Carattere Scientifico Neuromed, Pozzilli, 86077 Isernia, Italy; 11 Department of Experimental and Clinical Medicine, Magna Graecia University, 88100 Catanzaro, Italy; dtorella@unicz.it

**Keywords:** mitochondria, cAMP, AKAP1, AKAPs, protein kinase A, ROS

## Abstract

Second messenger cyclic adenosine monophosphate (cAMP) has been found to regulate multiple mitochondrial functions, including respiration, dynamics, reactive oxygen species production, cell survival and death through the activation of cAMP-dependent protein kinase A (PKA) and other effectors. Several members of the large family of A kinase anchor proteins (AKAPs) have been previously shown to locally amplify cAMP/PKA signaling to mitochondria, promoting the assembly of signalosomes, regulating multiple cardiac functions under both physiological and pathological conditions. In this review, we will discuss roles and regulation of major mitochondria-targeted AKAPs, along with opportunities and challenges to modulate their functions for translational purposes in the cardiovascular system.

## 1. Introduction

β1-adrenergic receptors (β1ARs) are members of the superfamily of heptahelical transmembrane receptors (7TMR), also known as G-protein-coupled receptors (GPCRs), and crucial regulators of multiple cardiovascular functions [1,2,3]. β1AR ligand binding induces receptor conformational changes that lead to the activation of G proteins and G-protein-mediated signaling, as well as G-protein-independent downstream effectors [4,5]. Stimulation of Gs by β1ARs increases intracellular levels of cyclic adenosine monophosphate (cAMP). cAMP rise induced by different agonists can produce different physiological responses, even within the same tissue, depending on the spatial and temporal regulation of the cAMP gradient by adenylyl cyclases (AC), phosphodiesterases (PDEs) and the local activation of effectors, mainly represented by the “classical” cAMP-dependent protein kinase A (PKA), cyclic nucleotide-gated ion channels and the more recently discovered exchange protein directly activated by cAMP (Epac) [6,7,8].

PKA holoenzyme is composed of two regulatory (R) and two catalytic (C) subunits, anchored to membranes and discrete cellular locations by a large family of A kinase anchor proteins (AKAPs) that play a crucial role in the propagation of cAMP/PKA signals. By binding PKA regulatory subunits (R) and specific intracellular organelles, AKAPs act as scaffolds that integrate and direct signaling events to downstream targets to achieve efficient spatial and temporal control of their phosphorylation state [9,10,11]. While the majority of AKAPs selectively bind PKA type II regulatory subunits (RII), dual-specificity AKAPs (D-AKAPs) bind to type I regulatory subunits (RI) and RII, potentially engaging distinct cAMP-responsive holoenzymes to specific intracellular locations. According to the cell type and pathophysiological context, the modulation of local signaling by AKAPs, either enhancement or disruption, transient or permanent, may regulate cardiovascular physiology, and its derangement may lead to pathological conditions. Restoring AKAP-regulated pathways may, thus, represent a novel approach for targeted therapy in cardiovascular diseases [12,13]. Several AKAPs have been identified in adult cardiac myocytes and localized at distinct subcellular compartments, including the nucleus, mitochondria, endoplasmic reticulum, sarcomere and others [11,14]. Their roles have been intensely investigated under physiological and pathological conditions over the last few years and described extensively in recent reviews [15,16].

## 2. Mitochondrial AKAPs

**Figure 1:**
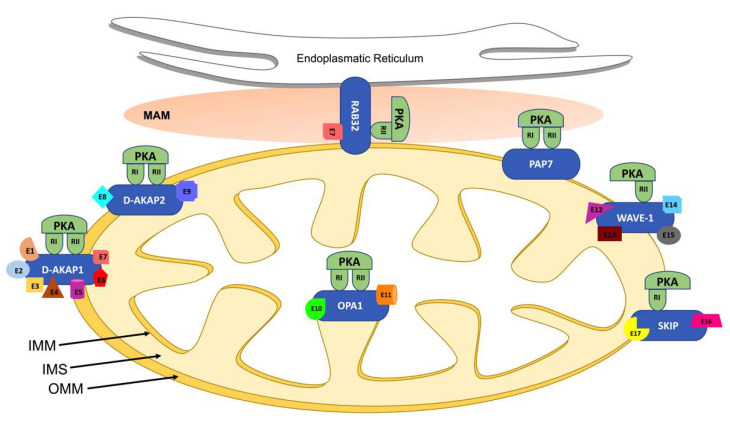
Mitochondrial AKAPs localization. Schematic representation of mitochondrial AKAPs localization as discussed in the text. Abbreviations used: endoplasmic reticulum, ER; intermembrane space, IMS; outer mitochondrial membrane, OMM; inner mitochondrial membrane, IMM; mitochondria-associated membranes, MAM; different binding effectors partners, E.

Mitochondria represent the most important cellular power generators, and their activity is involved in the regulation of many functions, including cell survival, death, metabolism, calcium homeostasis and production of reactive oxygen species (ROS). Mitochondrial dynamics, the regulated balance of fusion and fission, represents a key aspect of mitochondrial function and is implicated in pathological conditions, including cardiovascular disorders such as cardiac hypertrophy, cardiac arrhythmias and heart failure [17,18]. The cAMP/PKA pathway has been previously found to regulate several mitochondrial functions, such as respiration, mitochondrial dynamics, ROS production and apoptosis [19]. cAMP signals are carried to mitochondria by several AKAPs, including D-AKAP1 (encoded by the Akap1 gene), D-AKAP2 (Akap10 gene), PAP7 (Acbd3 gene), OPA1 (Opa1 gene), WAVE-1 (Wasf gene), RAB32 (Rab32 gene) and SKIP (SPHKAP gene), summarized in Table 1 and schematically illustrated in Figure 1 [20,21,22]. Recent biochemical and immunocytochemical studies indicate that D-AKAP1, D-AKAP2 and ACBD3 are the predominant mitochondrial AKAPs exposed to the cytosolic compartment in adult rat ventricular myocytes; thereby, both RI and RII PKA subunits are associated with mitochondria [21,23,24].

### 2.1. D-AKAP1

D-AKAP1 is a mitochondrial AKAP encoded by the Akap1 gene and expressed in various organs and cell types, including the cardiovascular system, and it is highly expressed in the human heart [12,16,21]. AKAP121, AKAP149 and the smaller variant s-AKAP84 are prototypic members of the D-AKAP1 family, sharing a similar 525-aminoacid NH2-terminal core but diverging significantly at the C-terminus. PKA binding domains of the rat, mouse and human D-AKAP1 homologues are highly conserved and bind PKA RII subunits with high affinity and RI with lower affinity. The first 30 NH2-terminal residues mediate the targeting of D-AKAP1-PKA complexes to the outer mitochondrial membrane (OMM, Figure 1). Within this motif, AKAP149, AKAP121 and S-AKAP84 present a conserved tubulin-binding motif, allowing interaction with both tubulin polymers and soluble microtubules [44]. Although D-AKAP1 has been primarily localized at the OMM, some variants have also been localized intracellularly at other intracellular locations, including the nuclear membrane and the endoplasmic reticulum. In addition to the PKA-binding domain and mitochondria-targeting domain, some members of the D-AKAP1 family also present a K homology domain (KH), enabling binding of AU-rich RNAs or single-strand DNA [12,45].

Accumulation of D-AKAP1 mRNA is positively regulated by cAMP/PKA signaling. Neonatal cardiomyocytes and vascular smooth muscle cells show a significant increase in D-AKAP121 mRNA and protein levels after incubation with βARs agonist isoproterenol, and this effect is abolished by pre-treatment with PKA inhibitors [46,47].

In addition to transcriptional regulation, D-AKAP1 undergoes rapid ubiquitination and proteosomal degradation upon hypoxia, mediated by the E3 ubiquitin ligase seven in-absentia homologue (Siah2) [48]. Since D-AKAP1 controls mitochondria dynamics through PKA-dependent inhibitory phosphorylation of Dynamin-related protein 1 (Drp1) and PKA-independent inhibition of Drp1–Fis1 interaction, its reduced availability mediated by Siah2 relieves Drp1 inhibition, increasing its interaction with Fis1, thus resulting in mitochondrial fission [49].

A multivalent signaling complex nucleated by D-AKAP1 on the OMM that includes, among other molecules, PKA, tyrosine-protein kinase Src, serine/threonine protein phosphatase 1 (PP1), cyclic nucleotide phosphodiesterase 4 (PDE4), Drp1, calcineurin, Na^+^/Ca^2+^ exchanger NCX3 and argonaute 2 protein (Ago2), has been previously identified as an essential regulator of multiple mitochondrial functions and extensively reviewed elsewhere [12,50,51]. Several data also indicate that D-AKAP1 anchors additional components, including the ribonucleoprotein granule components La-related protein 4 (LARP4), polyadenylate-binding protein 1 (PABPC1) and mRNAs encoding proteins required for mitochondrial metabolism. Thus, loss of D-AKAP1–RNA interactions might also impair mitochondrial electron transport chain activity [52,53]. D-AKAP1 also facilitates the function of PTEN-induced kinase 1 (PINK1), rapidly degraded in healthy mitochondria but selectively stabilized on the OMM of defective mitochondria to initiate mitophagy and modulate other processes, including mitochondrial fission and mobility [54].

#### 2.1.1. D-AKAP1 in Cardiovascular System and Metabolism

D-AKAP1 knockout mice have been generated by deletion of the first coding exon, which includes the mitochondrial targeting sequence and the PKA binding site [55]. Mice with homozygous or heterozygous global genetic D-AKAP1 inactivation (Akap1^−/−^ or Akap1^+/−^, respectively) display normal cardiac structure or function, cardiomyocyte size or fibrosis compared to their wild-type littermates [25]. However, under basal conditions, Akap1^−/−^ mice display remarkable abnormalities in mitochondrial structure at electron microscopy [26].

Both in cardiomyocytes and in several murine models of cardiovascular disease, D-AKAP1 deficiency impairs mitochondrial structure and respiratory function, reduces ATP production and increases cardiomyocyte apoptosis via enhanced mitochondrial ROS production [25,26,27]. Moreover, D-AKAP1 expression is reduced in hearts of streptozotocin-induced diabetic mouse models, and its genetic inactivation significantly enhances cardiac dysfunction in diabetic mice. Immunoprecipitation and mass spectrometry indicate that D-AKAP1 interacts with the NADH-ubiquinone oxidoreductase 75 kDa subunit (NDUFS1) in the heart, and, therefore, D-AKAP1 might also modulate complex I activity by regulating NDUFS1 translocation from cytosol to mitochondria [56]. Indeed, restoration of D-AKAP1 expression in the hearts of streptozotocin-treated diabetic mice promotes NDUFS1 translocation to mitochondria and ameliorates diabetic cardiomyopathy [56].

Compared to wild type, Akap1^−/−^ mice of either sex display a mild but significant increase in systolic blood pressure levels even if within the normal range, while Akap1^+/−^ mice display an intermediate phenotype. These differences can be largely attributed to a selective impairment in endothelium-dependent vasorelaxation in mice with partial or total loss of D-AKAP1 [25]. Indeed, D-AKAP1 inactivation also profoundly alters mitochondrial structure and function of vascular endothelial and smooth muscle cells, reducing mitochondrial membrane potential, increasing mitochondrial ROS production and, finally, enhancing hypoxia-induced cell dysfunction or death [26,27]. In particular, Akap1^−/−^ endothelial cells display remarkable mitochondrial alterations impacting multiple in vitro and in vivo functions, such as proliferation, migration and differentiation [25]. After femoral artery ligation, D-AKAP1 levels are rapidly and persistently reduced in skeletal muscles of ischemic limbs [25]. Compared to wild-type mice, Akap1^−/−^ mice exhibit impaired blood flow and delayed functional recovery after hindlimb ischemia, consistent with reduced post-ischemic hindlimb neovascularization. Moreover, Akap1^−/−^ ischemic limb muscles display a significant reduction in capillary density, increased fibrosis and marked mitochondrial abnormalities, and these pathological signs are mostly prevented in Siah2^−/−^ mice [25].

#### 2.1.2. D-AKAP1 and Myocardial Infarction

D-AKAP1 also plays a protective role in myocardial ischemia, regulating mitochondrial structure and function, ROS production and cell survival [26]. Upon in vitro hypoxia, cells lacking Siah2 display higher D-AKAP1 levels, and this is associated with reduced fission and apoptosis [49]. After coronary artery ligation to induce myocardial infarction (MI), Akap1^−/−^ mice display remarkable abnormalities in mitochondrial structure, increased cardiomyocyte death and infarct size, aggravating cardiac [57] remodeling and accelerating HF development. Genetic inactivation of Siah2 prevents D-AKAP1 degradation after MI, reducing infarct size and ameliorating cardiac remodeling and survival [26].

#### 2.1.3. D-AKAP1 and Cardiac Hypertrophy Induced by Pressure Overload

Cardiac hypertrophy is the first general response of the heart to physiological or pathological loads [58,59]. We and others have shown that pathological stress is able to specifically activate detrimental signaling pathways in the heart [60]. Among these, abnormalities in βAR signaling are the best studied and characterized, seem to be mechanistically linked to the development of cardiac dysfunction [60] and are hallmarks of the failing human heart [61]. We have previously shown that D-AKAP1 signaling is impaired in pathological cardiac hypertrophy induced by pressure overload in the rat, leading to mitochondrial dysfunction [57]. In response to pressure overload, cAMP response element-binding protein (CREB) phosphorylation is rapidly and significantly inhibited, altering PKA-dependent transcription of CREB-induced genes, including D-AKAP1. Thus, D-AKAP1 down-regulation may be a direct consequence of this block since its transcription is dependent by cAMP [46]. In addition, more recent data indicate that D-AKAP1 is also subjected to ubiquitin-dependent degradation [48], and this may be operating in the early phases of cardiac stress, such as acute myocardial ischemia. Eventually, transcriptional silencing of the Akap1 gene might be responsible for the long-term or persistent loss of the protein at mitochondria. D-AKAP1 downregulation is associated with marked abnormalities in mitochondrial morphology and structure at electron microscopy, reduced aconitase activity and increased mitochondrial ROS generation, suggesting that D-AKAP1 is an important regulator of mitochondrial function and ROS generation in the overloaded heart [57].

Loss of D-AKAP1 expression is also associated with reduced phosphorylation and nuclear localization of transcription factor nuclear factor of activated T-cells (NFAT), a downstream effector of the pro-hypertrophic phosphatase calcineurin, while its overexpression in cardiac myocytes blocks hypertrophy induced by isoproterenol [62]. Consistent with these results, in vitro mitochondrial competitive displacement of D-AKAP1 from mitochondria is able to induce NFAT nuclear translocation even though the short-term in vivo administration of these peptides does not induce cardiac hypertrophy [57]. It is possible to speculate that cardiac hypertrophy requires a prolonged treatment with D-AKAP1 displacing peptides or that ‘delocalized’ pools of D-AKAP1 in vivo might still bind and sequester calcineurin, thus preventing the activation of the NFAT-dependent hypertrophic gene program.

After one week of transverse aortic constriction [63], Akap1^−/−^ mice display enhanced cardiomyocyte and left ventricle hypertrophy and accelerated progression towards heart failure (HF) compared to wild type mice. This phenotype is associated with a significant increase in cardiac apoptosis as well as a lack of activation of Akt signaling after pressure overload [27]. Taken together, these results suggest that in vivo genetic ablation of D-AKAP1 promotes pathological cardiac hypertrophy and HF, indicating D-AKAP1 as a novel repressor of pathological left ventricular remodeling and failure.

### 2.2. D-AKAP2

D-AKAP2 is encoded by the Akap10 gene, mainly expressed in brain, skeletal muscle, kidney and testis, wherein it anchors PKA on the OMM (Figure 1). D-AKAP2 consists of 372 amino acids, binding PKA, RI and RII subunits at its C-terminus, containing two domains for G-protein regulation (RGS) and one PDZ domain to anchor membrane proteins [64,65]. Through the RGS domain, D-AKAP2 interacts with Rab4 and Rab11 GTPases, regulating vesicular endocytic trafficking by exposure of membrane receptors, ligands and lipids [64]. D-AKAP2 is also involved in the production of red blood cells since it induces GATA1 signaling pathway activation during erythropoiesis [66].

#### 2.2.1. D-AKAP2 and Cardiovascular Function

As other dual AKAPs, D-AKAP2 is capable of binding both RI and RII subunits of PKA in cardiac cells under physiological conditions. Synthetic peptides spanning the PKA binding domain of D-AKAP2, by inhibiting the PKA/D-AKAP2 complex formation, exert negative effects on cardiac chronotropy, inotropy and lusitropy, suggesting a key role for D-AKAP2-mediated targeting of PKA in the control of heart rate and contractile function [28].

#### 2.2.2. D-AKAP2 and Myocardial Infarction

D-AKAP2 knockout in cardiomyocytes of adult mice increases infarct size and accelerates HF induced by MI, as shown by LV enlargement and reduced function [29], since, in cardiomyocytes, D-AKAP2 promotes PKA-mediated activation of the steroid receptor co-activator 3 (Src3) and estrogen receptor α (ERα). As expected, cardiomyocyte-specific D-AKAP2 knockout decreased the transcription of ER-dependent genes involved in cell survival and angiogenesis, including Bcl2 and vascular endothelial growth factor a (VEGFa), thus blunting pro-angiogenic and anti-apoptotic signals in the ischemic heart [29]. Moreover, several polymorphisms of the AKAP10 gene are associated with cardiac dysfunction. In particular, the A→G (Ile646Val) polymorphism is associated with an increased risk of myocardial infarction in patients without hypercholesterolemia and in patients without hypercholesterolemia and high levels of serum HDL-cholesterol [67].

#### 2.2.3. D-AKAP2 and Cardiac Arrhythmias

D-AKAP2 acts as a controller of pacemaker cells sensitivity to cholinergic stimulation, both in mouse-embryonic-stem-cell-derived cardiomyocytes and, in vivo, in murine hearts. Consistent with these data, Akap10-deficient mice display heart rhythm abnormalities and suffer sudden death [30]. In physiological conditions, D-AKAP2 is able to regulate the activity of potassium channels (GIRK) by interacting with G protein, while, in Akap10-deficient mice, the regulation of the GIRK channels by D-AKAP2 and G proteins is impaired, resulting in sinus arrhythmia. Furthermore, the polymorphism Ile646Val affecting the affinity of D-AKAP2 for the regulatory subunit RI of PKA has been associated with increased basal heart rate and decreased heart rate variability, both effects potentially increasing the risk of sudden cardiac death [30].

### 2.3. ACBD3/PAP7/GCP60

Acyl-coenzyme A binding domain containing 3 (ACBD3) was first identified as a Golgi-localized protein and also known as peripheral-type benzodiazepine receptor and cAMP-dependent protein-kinase-associated protein 7 (PAP7), Golgi complex-associated protein of 60 kDa (GCP60), Golgi complex-associated protein 1 (GOCAP1) and Golgi phosphoprotein 1 (GOLPH1). Even if generally associated with the Golgi apparatus, ABCD3 is also localized in the endoplasmic reticulum (ER), plasma membrane, cytosol and mitochondria [21,68]. At these locations, ACBD3 participates in multiple protein–protein interactions and has various functions, including regulation of steroidogenesis, embryogenesis, neurogenesis, membrane trafficking, viral/bacterial replication and iron uptake [31]. So far, very little is known regarding ABCD3 functions in the heart.

### 2.4. OPA1

Optic atrophy 1 (OPA1) is a recently identified AKAP localized at the inner mitochondrial membrane (IMM) [69], wherein it binds the PKA substrate coiled-coil helix coiled-coil helix domain-containing protein 3 (ChChD3) [70] and stabilizes mitochondrial cristae, increases mitochondrial respiratory efficiency and reduces mitochondrial dysfunction, cytochrome c release and ROS production [71]. In adipocytes, OPA1 assembles on the surface of lipid droplets a complex containing PKA and perilipin, a protein regulating lipases access to stored triglycerides, thus mediating adrenergic control of lipolysis [72].

Under physiological conditions, OPA1 is regulated by two peptidases—OMA1 and YME1L—which coordinate mitochondrial fission and fusion and converting long-OPA1 (L-OPA1) into short-OPA1 (S-OPA1). S-OPA1 and L-OPA1 control, respectively, mitochondrial fission and fusion, the unbalance of the two isoforms inducing mitochondria fragmentation [32]. In cardiomyocytes, deletion of Yme1l gene induces OMA1 activation, resulting in L-OPA1 and S-OPA isoforms unbalance induced by greater conversion of L-OPA1 into S-OPA1, increased mitochondrial fission and inhibition mitochondrial fusion. Moreover, in mice models with specific cardiac Yme1l deletion, increased cardiac dysfunction and fibrosis and a metabolic shift with increased glucose uptake and decreased acetyl-carnitine oxidation are observed, with consequent reduction in beta-oxidation. Double deletion of both Yme1l and Oma1 genes restores mitochondrial morphology and cardiac function [32].

In murine hearts after myocardial ischemia and reperfusion injury, the expression of OPA1 is downregulated, with a consequent decline in cardiomyocyte survival and mitochondrial function [73]. In contrast, OPA1 overexpressing mice show a reduction in heart and brain damage after cardiac ischemia and reperfusion [74]. In response to pressure overload, TNFα receptor 2 (TNFR2) activation induces OPA1 upregulation, with consequent reactivation of mitochondria dynamics and ATP recovery [75].

Cardiomyocytes from mice with partial deletion of the Opa1 gene (Opa1^+/−^) show an alteration in mitochondrial morphology by size and structure of the crests, i.e., larger mitochondria and incomplete mitochondrial fusion. Furthermore, after pressure overload, Opa1^+/−^ mice exhibit cardiac hypertrophy and myocardial dysfunction [33]. Consistent with these results, 12-month-old Opa1^+/−^ mice show reduced cardiac function and increases in ROS production and mitochondrial dysfunction [76].

### 2.5. WAVE-1

WAVE-1 is one of the Wiskott Aldrich syndrome proteins (WASP) family, and it has been previously implicated in transduction of signals from cell surface membrane receptors (ion channels, adhesion receptors, 7TMRs) to the actin cytoskeleton by binding RII-PKA [77,78]. WAVE 1 is very abundantly expressed in brain tissue, while its levels are extremely low in the other tissues, including the heart [79]. In normal conditions, WAVE1 is associated to actin in the cytoskeleton and coordinates actin dynamics [34]. Following an ischemic event in the brain, a rearrangement of the actin occurs, which places WAVE1 near the OMM of mitochondria to form a complex with pancortin-2 and Bcl-xL, and this association is critical for Bax binding and mitochondrial membrane pore formation for cytochrome c release [34]. Mice with WAVE1 deletion show a learning disability and loss of memory due to spine morphology dysfunction, and phosphorylation/dephosphorylation of WAVE1 is crucial for the regulation of dendritic spine morphology [35]. Moreover, WAVE1 mediates the anchoring of glucokinase (GK) and BAD on mitochondria to coordinate glucose metabolism and apoptosis [36,37]. The role of WAVE-1 in the heart is still largely unknown.

### 2.6. RAB32

Ras-related protein RAB32 (RAB32) is a member of the Ras superfamily of small molecular weight G-proteins, originally identified by yeast two-hybrid screens, binding RII of PKA [80]. RAB32 is expressed in heart, skin, spleen, liver and testis tissues [81], wherein it localizes at the mitochondria-associated membrane (MAM), space between the endoplasmic reticulum (ER) and mitochondria [81]. RAB32 regulates MAM membrane activity through the modulation of Ca^2+^ handling, mitochondria dynamics and apoptosis [80,81,82]. RAB32 anchors PKA and promotes Drp1 phosphorylation on Serine 656, in turn inactivating Drp1, inhibiting apoptosis and promoting mitochondrial fusion [38,81]. In addition, RAB32 has been involved in mitochondrial movements within neurites [39] and mitochondrial uptake of cytoplasmic proteins during ageing [40]. A recent study also localizes RAB32 in lysosomes, wherein it regulates metabolism and cell growth by the mTORC1 pathway [38]. Moreover, mice with genetic Rab32 and Rab38 depletion display impaired platelet function [41]. Currently, the role of RAB32 in the cardiovascular system is still largely unknown.

### 2.7. SKIP

Sphingosine kinase type 1-interacting protein (SKIP, SPHKAP) was identified by proteomics screens of cAMP interactome in mammalian heart tissues as an AKAP preferentially binding to PKA-RI, enriched at the IMM, wherein it associates with a prominent PKA substrate, the protein ChChD3 [83]. Immunoprecipitation studies demonstrate that SKIP is associated with sphingosine kinase type 1 (SPHK1), an important regulator of sphingolipid metabolism. SPHK1 phosphorylates sphingosine to form sphingosine-1 phosphate (S1P), which exerts several functions, including regulation of cell growth, survival and death [84]. Northern blot assay demonstrates that SKIP is expressed mainly in the heart but also in the spleen, ovary and brain [85]. SKIP expression can also be found in pancreatic β-cells, and mice with genetic SKIP depletion display increased glucose-stimulated insulin secretion under high glucose conditions [42]. Mouse models with global genetic depletion of SKIP, subjected to myocardial ischemia and reperfusion injury, show increased infarct size, apoptosis and cytochrome c release [43]. Interestingly, the interaction between PKA and SKIP was markedly increased in human heart failure samples [13].

## 3. Mitochondrial AKAPs as Novel Therapeutic Targets

**Figure 2:**
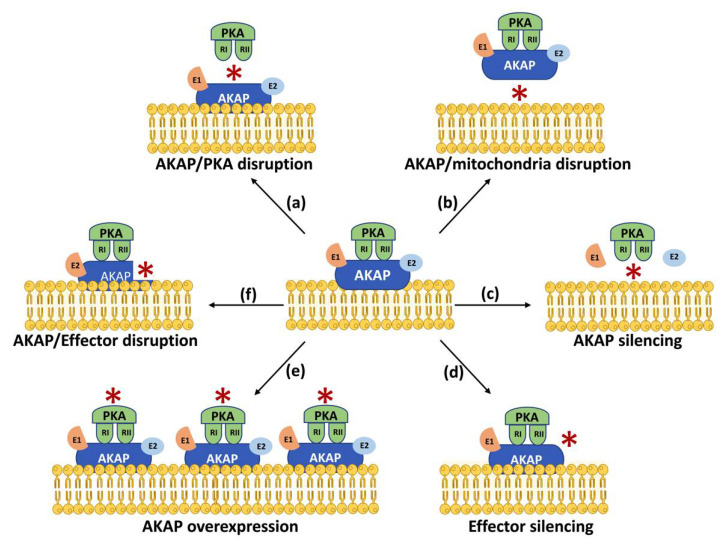
Strategies to modulate AKAP signaling. Schematic representation of different strategies to modulate AKAPs signaling: (**a**) disruption of AKAP/PKA interaction; (**b**) disruption of AKAP/mitochondria interaction; (**c**) silencing of effector molecules; (**d**) AKAP silencing; (**e**) AKAP overexpression and (**f**) disruption of AKAP/effectors complexes. Red asterisk (*) indicates the site of modulation.

Data discussed in this review highlight the crucial involvement of mitochondrial AKAPs in the regulation of several cardiovascular functions under physiological or pathological conditions, suggesting that these signaling hubs represent novel and druggable targets for multiple cardiovascular diseases. To obtain specific inhibition of mitochondrial AKAPs in the cardiovascular system, several aspects should be considered. Since AKAPs lack intrinsic enzymatic activity and function as nucleators of signalosomes at specific cellular locations—in some cases with still unknown cardiovascular functions—several different strategies could be potentially undertaken to modulate AKAP levels, mitochondrial targeting and/or selective interactions with effectors (Figure 2). While the use of global activators or inhibitors of cAMP/PKA signaling is expected to indiscriminately affect all compartmentalized intracellular pools of cAMP, overexpression or silencing of specific AKAPs with a demonstrated mechanistic role in specific physiological or pathological conditions may represent valuable approaches to restrict spatial regulation of AKAPs signaling [8]. Signalosomes nucleated by AKAPs usually involve multiple proteins complexes, and their balanced levels might be required for specific cellular functions. A further possibility to interfere with protein–protein interactions in mitochondrial AKAPs signalosomes is the selective silencing of AKAPs or their relative targets. The observed effects can be variable according to the modulated AKAP, the effector, the cell type and the experimental conditions. For example, D-AKAP1 silencing has been shown to promote cardiomyocyte hypertrophy and apoptosis [62], while silencing of OPA1, an emerging key molecule in cancer cell biology, has been shown to reduce cells’ proliferation and migration [86]. In addition, Opa1 expression has been shown to prevent cardiomyocyte apoptosis and sustain cardiomyocyte function during hypoxic stress by enhancing mitochondrial turnover and respiratory capacity [87]. Knockout studies have shown that D-AKAP2 regulates infarct size and HF development after MI as well as heart rhythm abnormalities and the risk of sudden death [29,30]. However, mitochondrial AKAPs might be required to preserve mitochondrial structure, function and/or dynamics under basal or stressful conditions, and, therefore, general modulation of their levels might determine unwanted effects.

Alternatively, gene editing of mitochondrial AKAPs might represent a novel strategy to assess the role of crucial domains, interacting effectors and their products in mitochondrial function, structure, metabolism and, ultimately, pathobiology. Indeed, specific AKAPs polymorphisms/mutations have been correlated to the risk of several cardiovascular diseases, but the precise mechanisms underlying these associations are still largely unknown [88].

The possibility to interfere with AKAP/PKA binding or AKAP mitochondrial localization might represent other possible therapeutic approaches to modulate AKAP-mediated signaling. RI- or RII-selective disruptors of AKAP/PKA complexes designed according to the known sequences of specific AKAPs have been previously described and reviewed [89,90]. Given the remarkable conservation of these sequences, these molecules are not very specific and can barely differentiate between type I and type II PKA [89]. Single nucleotide polymorphisms of AKAPs can affect their subcellular localization [91]. Alternatively, peptides can be designed to competitively displace AKAPs from specific intracellular locations, including mitochondria. Given the specific targeting of mitochondrial AKAPs to OMM, IMM, IMS and MAM (Figure 1), it would also be crucial to estimate the effects of specific molecular interventions on sub-mitochondrial localization and membrane topology of known mitochondrial proteins [92]. Although peptides present several limitations preventing their immediate translational application in vivo, including their short life, low cellular permeability and resistance to degradation, these drawbacks could be overcome by other small molecules or compounds with higher stability and permeability, with similar specificity.

We have previously demonstrated that competitive displacement of D-AKAP1 from mitochondria promotes mitochondrial dysfunction and increases ROS generation and cell death in rat neonatal cardiomyocytes and the whole heart [57]. These studies highlight the crucial role of D-AKAP1 in the heart and are particularly important considering that most AKAPs are expressed in multiple systems, organs and cell types, wherein they exert tissue- and cell-specific functions and, therefore, the effects of their inhibition in other—even remote—organs and tissues should always be considered. For example, high levels of D-AKAP1 have been identified in a wide variety of high-grade cancer tissues, wherein they correlate with cancer cells’ growth and patients’ survival, suggesting that D-AKAP1 silencing may represent a new target for therapy in cancer with possible unwanted cardiac side effects [93].

## 4. Conclusions

Accumulating evidence identifies subcellular cAMP signaling as the main contributor of cardiomyocyte homeostasis. As a consequence, disruption of microdomains of cAMP triggers cardiomyocyte dysfunction, promoting cardiovascular disease. With their role in regulating local cAMP signaling, mitochondrial AKAPs are uniquely positioned to modulate mitochondrial function and, in general, cellular bioenergetics in cellular health and disease. Hence, work to unravel mechanisms of mitochondrial AKAPs function holds great basic and, potentially, clinical potential.

## Figures and Tables

**Table 1:** Mitochondrial AKAPs and their known cardiovascular functions.

Gene	Protein	Effectors	PKA Binding	Mitochondrial Localization	Mitochondrial Effects	Cardiovascular Functions	References
Akap1	D-AKAP1	PP1(E1), Src(E2), PDE4(E3), Calcineurin (E4), AGO2(E5), NCX3(E6), DRP1(E7)	RI, RII	OMM	Apoptosis, mitochondrial fusion, mitochondrial respiration	Modulates infarct size after coronary artery ligation and post-ischemic cardiac remodelling, cardiac hypertrophy and pressure-overload-induced cardiac dysfunction, endothelial function and angiogenesis	[25,26,27]
Akap10	D-AKAP 2	Rab 4(E8), Rab11(E9)	RI, RII	OMM	Mitochondrial dynamics, ROS production and apoptosis	Modulates cardiovascular integrity barrier and controller of pacemaker cells’ sensitivity to cholinergic stimulation	[28,29,30]
Acbd3	PAP7		RI, RII	OMM	Cholesterol transport	Unknown	[31]
Opa1	OPA1	OMA1(E10), YME1L(E11)	RI, RII	IMM	Mitochondrial fusion and fission, stabilizing mitochondrial cristae, increasing mitochondrial respiratory efficiency	Modulates cardiac function and hypertrophy, metabolic shift of increased glucose uptake	[32,33]
Wasf	WAVE-1	BAD(E12), Pancortin2(E13), GK(E14), BCL-XL(E15)	RII	OMM	Mitochondrial trafficking	Unknown	[34,35,36,37]
Rab32	RAB32	DRP1(E7)	RII	MAM	Endoplasmic reticulum Ca^2+^ handling, mitochondrial fusion, apoptosis	Unknown	[38,39,40,41]
Sphkap	SKIP	ChChdl3(E16), S1P(E17)	RI	OMM	Mitophagy	Modulates infarct size, apoptosis and cytochrome c release after myocardial ischemia-reperfusion injury	[42,43]

Abbreviations: PKA, protein kinase A; RI, regulatory subunit type I; RII, regulatory subunit type II; OMM, outer mitochondrial membrane; IMM, inner mitochondrial membrane; MAM, mitochondria-associated membrane.

## Data Availability

Not applicable.
